# Extra-gastrointestinal stromal tumor of the greater omentum: report of a case and review of the literature

**DOI:** 10.1186/1477-7819-6-25

**Published:** 2008-02-23

**Authors:** Christian Franzini, Luciano Alessandri, Irene Piscioli, Salvatore Donato, Rosario Faraci, Luca Morelli, Franca Del Nonno, Stefano Licci

**Affiliations:** 1 Department of General Surgery, District Hospital of Guastalla (RE), Italy; 2 Department of Radiology, Hospital of Budrio (BO), Italy; 3 Department of Radiology, Hospital of Bentivoglio (BO), Italy; 4 Department of Pathology, "S. Maria del Carmine" Hospital, Rovereto (TN), Italy; 5 Department of Pathology, "National Institute for Infectious Diseases – L. Spallanzani" IRCCS, Rome, Italy

## Abstract

**Background:**

Gastrointestinal stromal tumors (GISTs) represent the majority of primary non-epithelial neoplasms of the digestive tract, most frequently expressing the KIT protein detected by the immunohistochemical staining for the CD117 antigen. Extra-gastrointestinal stromal tumors (EGISTs) are neoplasms with overlapping immunohistological features, occurring in the abdomen outside the gastrointestinal tract with no connection to the gastric or intestinal wall.

**Case presentation:**

We here report the clinical, macroscopic and immunohistological features of an EGIST arising in the greater omentum of a 74-year-old man, with a discussion on the clinical behavior and the prognostic factors of such lesions and a comparison with the gastrointestinal counterpart.

**Conclusion:**

The EGISTs in the greater omentum can grow slowly in the abdomen for a long time without clinical appearance. In most cases a preoperative diagnosis is not possible, and the patient undergoes a surgical operation for the generic diagnosis of "abdominal mass". During the intervention it is important to achieve a complete removal of the mass and to examine every possible adhesion with the gastrointestinal wall. Yamamoto's criteria based on the evaluation of the mitotic rate and the MIB-1 labelling index seems to be useful in predicting the risk for recurrence or metastasis. More studies are necessary to establish the prognostic factors related to localization and size of the EGIST and to evaluate the impact of the molecular characterization as an outcome parameter related to the molecular targeted therapy. In absence of these data, an accurate follow-up is recommended.

## Background

Stromal tumors represent the majority of primary non-epithelial neoplasms of the digestive tract and are collectively defined gastrointestinal stromal tumors (GISTs). They histologically, immunohistochemically and genetically differ from leiomyomas, leiomyosarcomas and schwannomas. GISTs may be defined as intra-abdominal mesenchymal tumors most frequently expressing the KIT protein, having a gain-of-function mutation in the regulatory juxtamembrane domain of the c-kit gene or an activating mutation in another class III receptor tyrosine kinase gene, the PDGFRA gene, which encodes the platelet derived growth factor receptor-alpha receptor tyrosine kinase protein [1,2]. The KIT protein can be detected by immunohistochemical assays for the CD117 antigen.

GISTs are most commonly found in the stomach (40 to 70%), small intestine (20 to 50%) and colorectum (5 to 15%) [3-6]. Neoplasms with histology and immunohistochemistry similar to GISTs may occur outside the gastrointestinal tract, for example in the soft tissue of the abdominal cavity (in particular omentum and mesentery) or in the retroperitoneum [7-9].

These tumors must be defined as extra-gastrointestinal stromal tumors (EGISTs) since they display no connection with the gastric or intestinal wall. While the histogenesis, prognostic parameters and outcomes of GISTs are widely known, pathogenesis, incidence and prognosis of EGISTs have not yet been completely defined. A comparison between GISTs and EGISTs is therefore of particular interest in order to understand whether they have a common cellular origin and a similar clinical behavior. We report the results of the macroscopic and microscopic examinations, including immunohistochemical studies, of an EGIST of the greater omentum. We discuss the clinical behavior and the prognostic factors through a review of the literature.

## Case presentation

A 74-year-old man was admitted to the Guastalla District Hospital in October 2005 because of a large abdominal mass. Five days before admission he was examined by his general practitioner because of sudden lower abdominal pain. Ultrasonography showed a nonhomogenous hypoechoic mass with multiple cystic components occupying almost all the superior abdomen.

**Figure 1:**
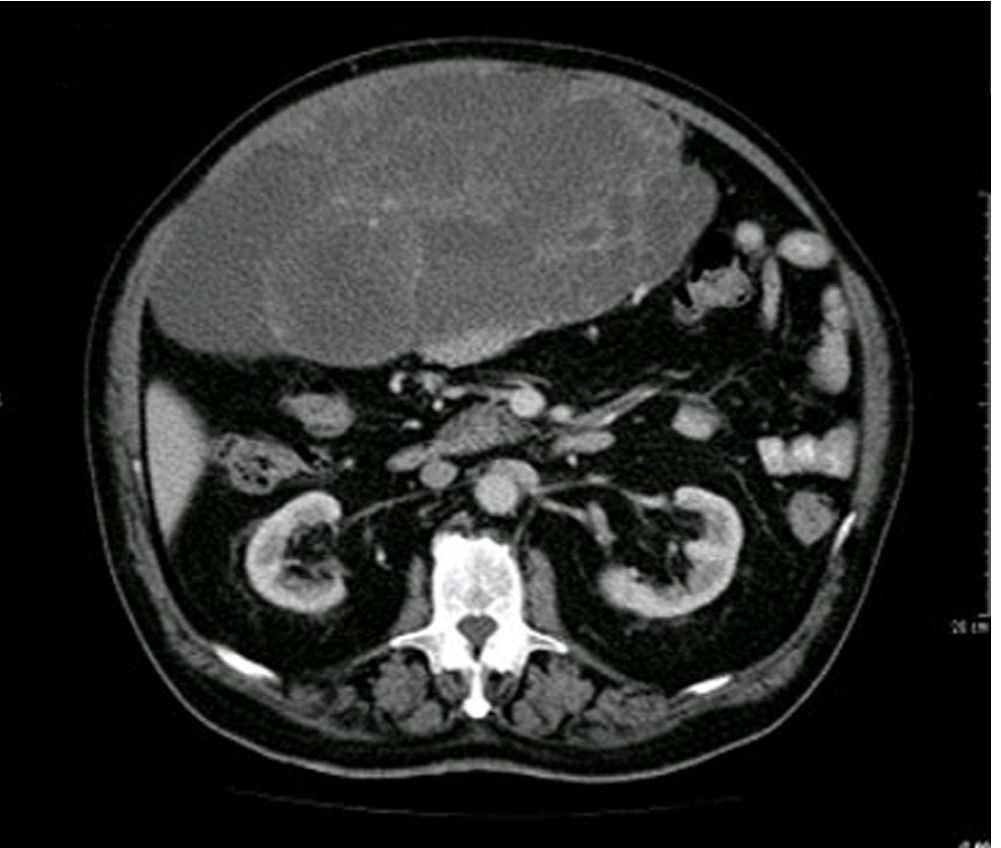
CT scan showing a voluminous intraperitoneal mass 33 × 30 × 17 cm in size, occupying the most part of the abdomen, with solid and cystic parts and with peripheral contrast enhancement.

Abdominal computed tomography (CT) (Figure 1) demonstrated a voluminous intraperitoneal mass, 33 × 30 × 17 cm in size, with cystic areas, solid parts and peripheral contrast enhancement. The bowel was dislocated without signs of intestinal occlusion. It was not possible to state with certainty the origin of the tumor.

Laparotomy revealed a large, slightly capsulated mass, arising from the greater omentum and the gastro-colonic ligament, without connection with the gastrointestinal tract. The mass was removed "en bloc" with the greater omentum and the gastro-colonic ligament. The tumor seemed completely excised. In order to achieve a radical omentectomy, the gastro-epiploic left and right vessels were ligated at their origin, and the greater gastric curvature and the transverse colon were skeletonized.

**Figure 2:**
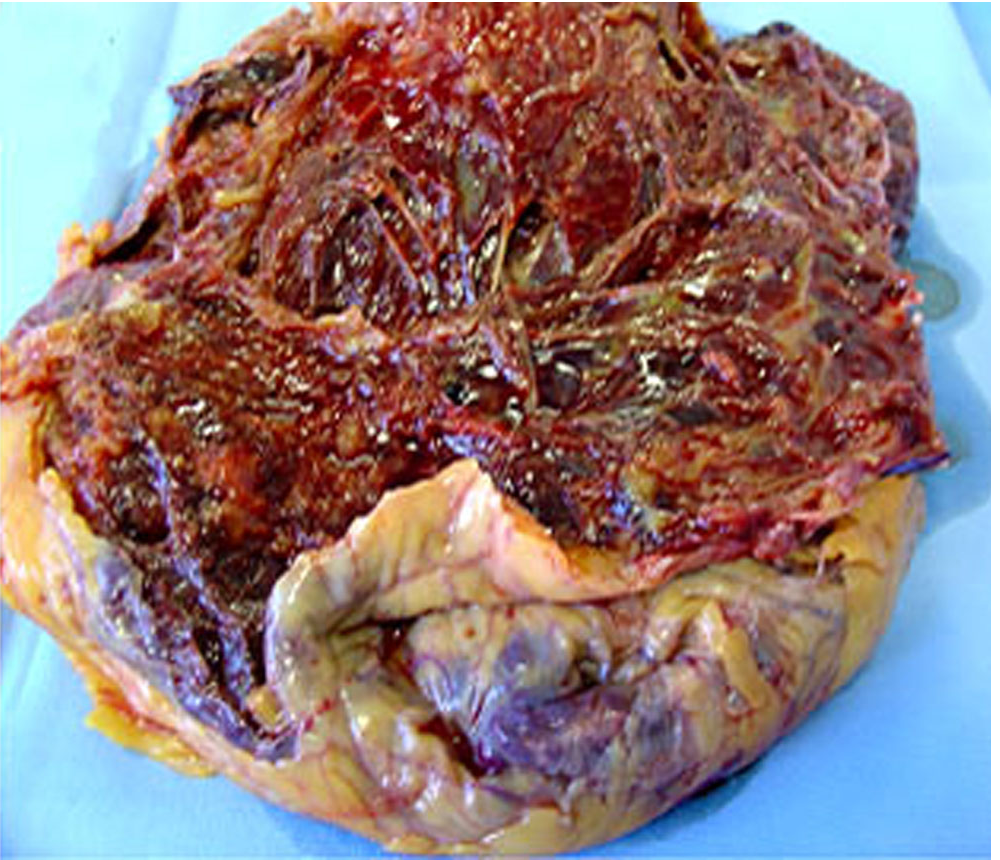
Gross appearance of the neoplastic mass, consisting of solid areas and cysts filled with clotted blood.

The tumor was 33 cm in maximum diameter and weighed 3500 g. On section the neoplasm consisted of whitish-grey and relatively firm areas and cystic areas filled with clotted blood (Figure 2).

**Figure 3:**
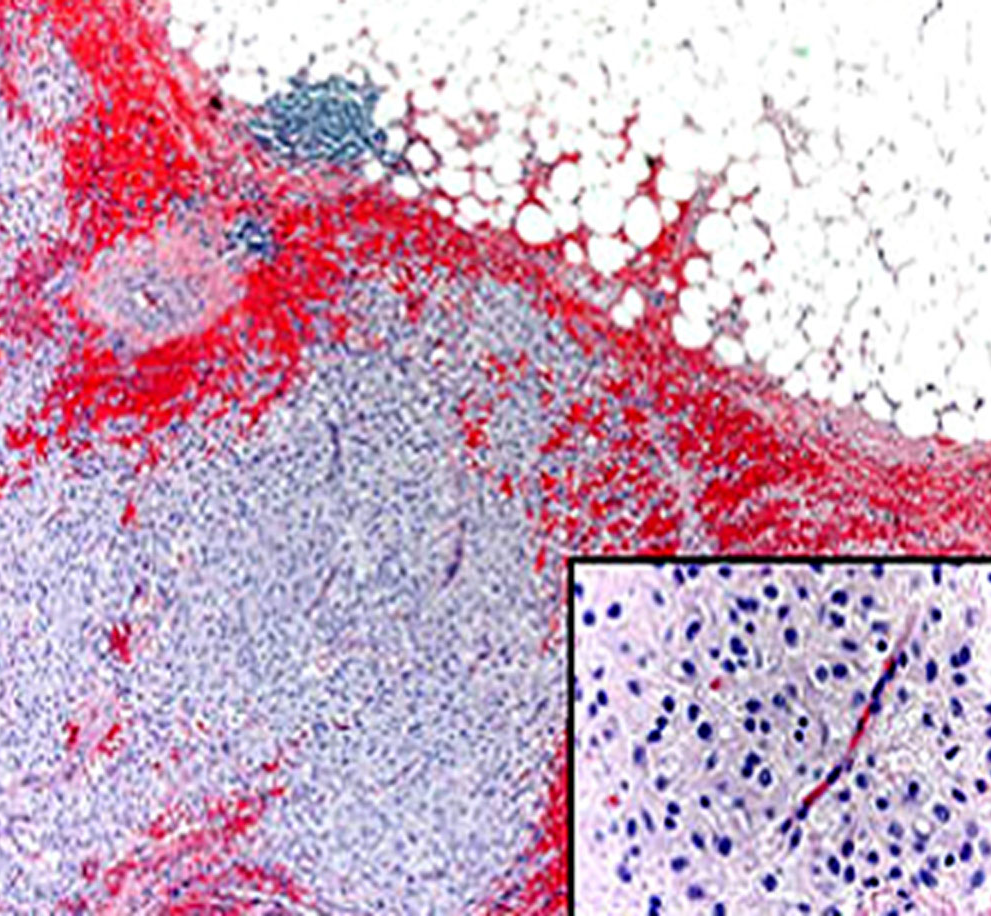
The tumor consists of sheets and aggregates of closely packed polygonal cell (hematoxylin and eosin, original magnification 100×), with abundant somewhat granular cytoplasm, as seen in the epitheliod type (inset, hematoxylin and eosin, original magnification 400×).

**Figure 4:**
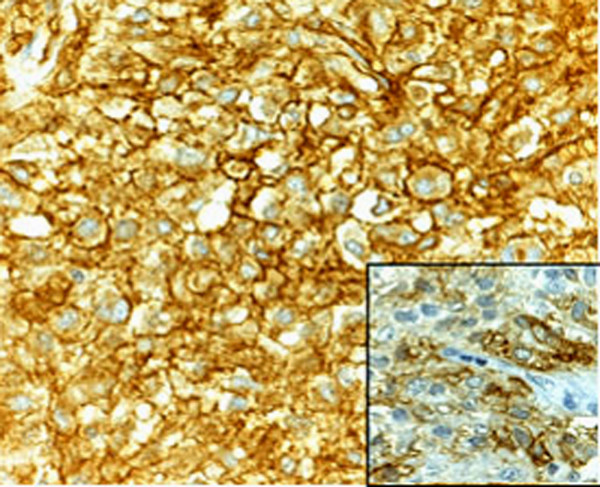
The neoplastic cells are immunoreactive for CD34 (original magnification 400×) and CD117 (inset, original magnification 200×).

Histologically the tumor consisted of closely packed polygonal cells, with abundant, somewhat granular cytoplasm arranged in sheets or dispersed singly throughout a finely collagenized background (Figure 3). Multinucleated cells were found. The mitotic activity was < 1 mitosis/50 high-power field (HPF). The MIB-1 labelling index was <10%. Extensive hemorrhage, foci of mixoid degeneration and focal necrosis were present. Immunohistochemical studies showed strongly positive staining of tumor cells for CD34 and CD117 (figure 4) and negative staining for desmin, smooth muscle actin (SMA), S-100 protein. These findings strongly supported a diagnosis of low risk EGIST of the greater omentum. A molecular genetic analysis for KIT protein mutation was not performed for its unavailability at our institute.

The patient had a regular hospital stay and was discharged eight days later. An abdominal CT showed no recurrence of disease 20 months after surgery.

## Discussion

EGISTs arise outside the gastrointestinal tract but they share histological features with their gastrointestinal counterpart. The clinical, pathological and prognostic features of GISTs are widely known, while data about EGISTs are very few: incidence, histogenesis and histological predictors of outcome are not yet defined.

EGISTs are rare tumors. Todoroki *et al*. [10] recently described one case citing 28 cases previously reported in the English-language literature. But the real incidence of this neoplasm could be lower. Agaimy *et al*. [11] revaluated 14 cases of EGISTs (four mesenteric, four omental, one pararectal, one pelvic, one perivescical, one of the mesenteric root, one involving the omentum and the abdominal wall and one located between liver and stomach). By means of a critical revaluation of the surgical reports and clinical histories and a careful search for residual muscular tissue from the gut wall in the tumor pseudocapsule, it was possible to reclassify most of these cases (11/14) either as GISTs with extramural growth or as metastases from a GIST. This study considered crucial the documentation by the surgeon during intervention of any attachment or adhesion, even minimal, to the gastrointestinal wall.

GISTs are currently considered as deriving from the interstitial Cajal cells (ICC). These are normally part of the autonomic nervous system of the intestine and they have a pacemaker function in controlling motility. Most GISTs (50–80%) arise because of a mutation in the c-kit gene. The c-kit/CD117 receptor is expressed on ICCs, mast cells, spermatocytes and hematopoietic cells. In the gut a tumor staining positive for CD117 is likely to be a GIST, arising from ICCs. The cell origin of EGISTs is controversial. Miettinen and Lasota [4] claim that omental and mesenteric EGISTs derive from stomach and small intestine respectively, representing tumors that, for some reason, have detached from their gastrointestinal original site during their development. In the study of 14 EGISTs arising from the omentum and mesentery by Li *et al*. [12], the multipotential mesenchymal stem cells are supposed to be the origin cell of these neoplasms.

Sakurai *et al*. [13] found the ICC-counterpart in the omentum. ICC-like cells were observed focally in the omentum at 21 weeks of human gestation, when ICC were present in the intermuscular space of the GI tract [14].

The prognostic factors indicating the malignant potential of GISTs include mitotic rate, tumor size and location. Currently, lesions that measure less than or are equal to 2 cm or which do not exceed five mitoses per 50 HPFs are thought to have a lower malignant and metastatic potential. GISTs with a large size (> 10 cm in diameter) or with a high mitotic count (> 10/50 HPFs) and GISTs with diameter >5 cm and more than 5 mitotic figures/50 HPFs are considered at high risk for recurrence [15].

In a study of more than 1000 GIST cases [5], subdivided into five locations (esophagus, stomach, small bowel, colorectum and peritoneum/mesentery/omentum), the tumor site seemed an independent prognostic factor. Esophageal tumors presented the most favorable prognosis, while peritoneal tumors had the lowest survival rate.

Recently, Wardelmann *et al*. [16] suggested to include the molecular data together with these classical prognostic parameters into the risk assessment of GISTs, since the molecular characterization is not only helpful for diagnostic purposes in cases with low or no KIT receptor expression but might also help to predict clinical prognosis as several subgroups with different risk of aggressive clinical behavior can be identified.

Predicting the potential biological behavior of the omental EGISTs remains difficult and the literature contains conflicting reports on this issue.

Miettinen *et al*. [8] examined nine cases of omental EGISTs and seven cases of mesenteric EGISTs. Omental EGISTs seemed to have a more favorable behavior, typically showing low mitotic counts, whereas mesenteric EGISTs appeared more aggressive (higher mitotic activity, frequent malignant behavior). No tumor-related deaths were documented during the follow-up in the nine patients with omental EGIST.

Reith et al. [17] have examined the clinico-pathological and immunohistochemical features of 48 EGISTs arising within the abdominal cavity (40 cases) and the retroperitoneum (remaining 8 cases). The tumors ranged in size from 2.1 to 32 cm with a median size of 12 cm and expressed CD117 (c-kit receptor) (100%), CD34 (50%), neuron-specific enolase (44%), SMA (26%), desmin (4%), and S-100 protein (4%). High cellularity, mitotic activity (>2 mitoses/50 HPF) and the presence of necrosis were significantly associated with an adverse outcome in univariate analyses, whereas nuclear atypia, growth pattern (spindled, epithelioid, or mixed) and size were not. The authors justified the finding of no association between tumor size and outcome of EGIST by the fact that the majority of EGISTs were large (>10 cm) when first detected. On the basis of the histological appearance and immunophenotypical profile the EGISTs seem to resemble stromal tumors originated from the small intestine rather than from the stomach.

Yamamoto et al. [7] examined the clinico-pathological features, prognostic factors, and c-kit and PDGFRA mutations in 39 cases of EGISTs including three omental tumors. These authors have defined three categories on the basis of a combination of the mitotic rate and MIB-1 labelling index: the high-risk group (>or=5/50 HPF with >or=10% Ki-67), the intermediate-risk group (>or=5/50 HPF with <10% Ki-67, or, <5/50 HPF with >or=10% Ki-67), and the low-risk group (<5/50 HPF with <10% Ki-67). The authors claim that the shortness of the follow-up period in the series by Reith et al. [17] (median: 24 months) may lead to a bias of their data. Moreover they pointed out that EGISTs were often large size due to their anatomic site, having enough space to grow and presenting clinical symptoms only after a long time. Therefore a grading system defined by a combination of mitotic rate and tumor size, which is commonly used in GISTs, may not be applicable in EGISTs. On the contrary, the molecular characterization of GISTs as a prognostic factor [16] can be expected to become in the next future a common prognostic parameter for such lesions, independently from the site of origin.

Surgery remains the standard treatment for non-metastatic EGIST in the greater omentum [18].

In our case the resection of tumor was complete and the neoplasm was considered at low risk for recurrence or metastasis according to Yamamoto's criteria. The accurate radiological follow-up (abdominal CT) has been considered the approach of choice in the control of the disease.

## Conclusion

The EGISTs in the greater omentum can grow slowly in the abdomen for a long time without clinical appearance and they are often referred to the surgeon when they have reached a large size. In most cases a preoperative diagnosis is not possible, and the patient undergoes a surgical operation for the generic diagnosis of "abdominal mass", that usually puts in apprehension both the patient and the surgeon. During the intervention it is important to achieve a complete removal of the mass, when possible "en bloc" with contiguous tissues and regional lymph nodes, even if prognostic value of lymphatic involvement of these tumors is still unclear. It is also crucial to examine every possible adhesion with the gastrointestinal wall, marking them for the pathologist. A histological diagnosis of EGIST is often unexpected. Yamamoto's criteria seem to be useful in predicting the risk for recurrence or metastasis. More studies are necessary to establish the prognostic factors related to localization and size of the EGIST and to evaluate the impact of the molecular characterization as an outcome parameter related to the molecular targeted therapy. In absence of these data, an accurate follow-up is recommended.

## Competing interests

The author(s) declare that they have no competing interests.

## Authors' contributions

CF, LA, IP, SD and RF participated equally in the design of the paper and in the study of the clinical and radiological data. LM, FDN and SL participated in the study of macroscopic and microscopic features of the lesion, in the design of the study and in the drafting of the manuscript. SL revised critically the final version of the manuscript. All authors read and approved the final manuscript.
